# Supplementary material for: Neurological Disorders Associated with WWOX Germline Mutations—A Comprehensive Overview
Source: Cells. 2021 Apr 7;10(4):824. doi: 10.3390/cells10040824 (PMC8067556; doi:10.3390/cells10040824)
Supplement: Supplementary file 1 [file cells-10-00824-s001.zip › supplementary materials/Supplemental Table Legends.docx]

**Table_1_SuppInfo: Published WWOX-related epilepsy patients-full table related to Table 1.** Summary of the genetic data (extended data), the number of cases and the seizure onset for 57 WOREE and 6 SCAR12 patients that were reported in more than 21 articles since 2014. 21 of the WOREE patients with deletion mutation, and most of the cases, 41, had seizures in the first two month after birth, and 1 case described seizures before birth.

**Table_2_SuppInfo: ClinVar variants of uncertain significance (VUS) that are linked to WOREE and SCAR12 patients.** Table includes patients with VUS of the *WWOX* gene that are less than 51 bp and linked to WOREE or SCAR12 deribved from ClinVar and compared with gnomAD databases. Most of these variants have not been reported in the literature in individuals with WWOX-related diseases and the available evidence is currently insufficient to determine the role of these variants in disease. Therefore, they have been classified as VUS.

**Table_3_SuppInfo: ClinVar pathogenic and likely pathogenic variants that are linked to WOREE and SCAR12.** Table contains pathogenic and likely pathogenic variants in the *WWOX* gene that are less than 51 bp and are linked to WOREE (EIEE28) or SCAR12. Some of these variants have not been reported in the literature in individuals with WWOX-related diseases but are predicted to cause loss of normal protein function.
