# Supplementary figures and images for: Neurological Disorders Associated with WWOX Germline Mutations—A Comprehensive Overview
Source: Cells. 2021 Apr 7;10(4):824. doi: 10.3390/cells10040824 (PMC8067556; doi:10.3390/cells10040824)

## Slide 1
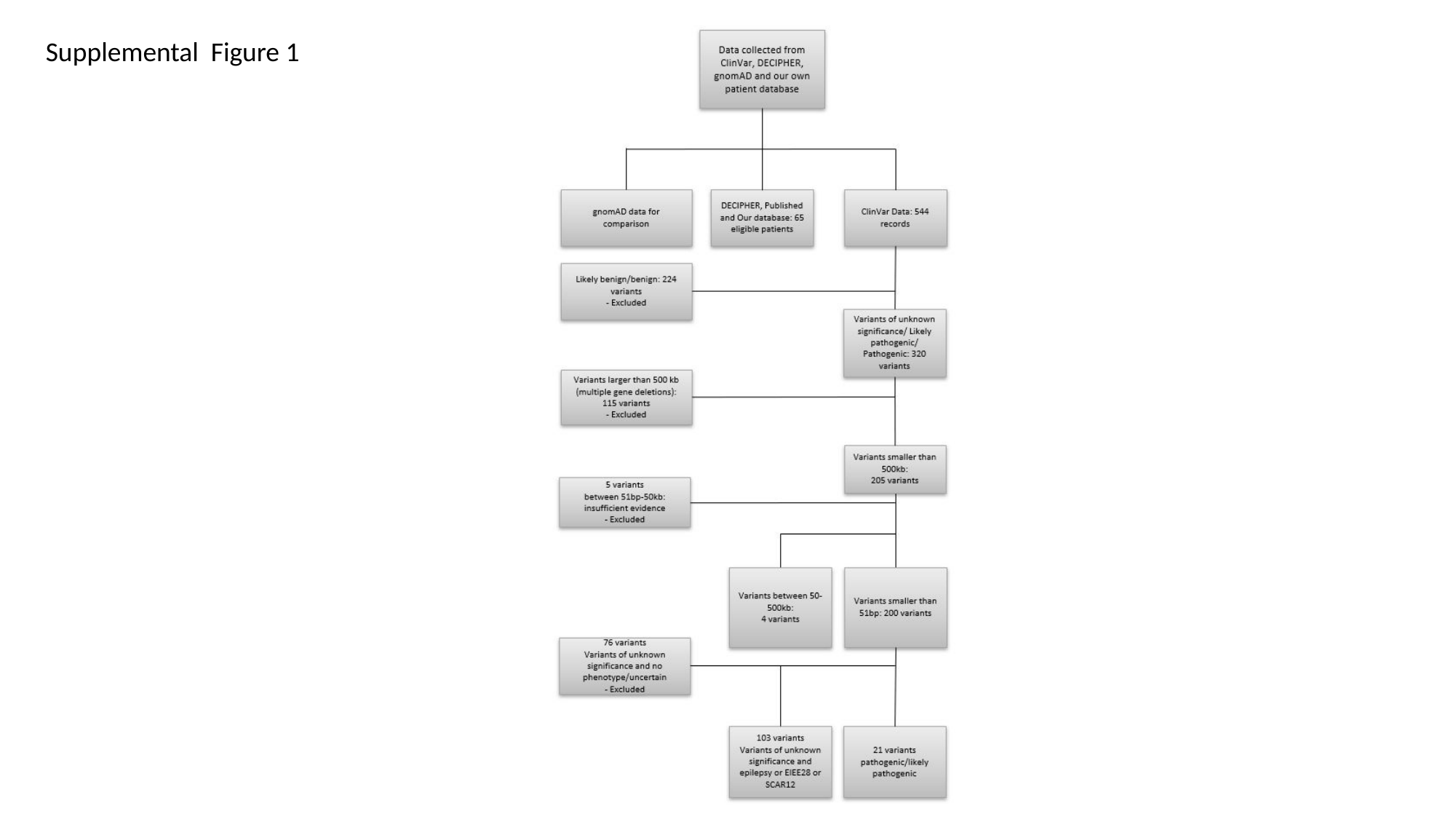

Supplemental Figure 1

Supplement: Supplementary file 1 [file cells-10-00824-s001.zip › supplementary materials/Supplemental Figure 1.pptx]
